# Antimicrobial, Insecticidal and Cytotoxic Activity of Linear Venom Peptides from the Pseudoscorpion *Chelifer cancroides*

**DOI:** 10.3390/toxins14010058

**Published:** 2022-01-14

**Authors:** Jonas Krämer, Tim Lüddecke, Michael Marner, Elena Maiworm, Johanna Eichberg, Kornelia Hardes, Till F. Schäberle, Andreas Vilcinskas, Reinhard Predel

**Affiliations:** 1 Institute of Zoology, University of Cologne, Zuelpicher Strasse 47b, 50674 Cologne, Germany; j.krae12@gmail.com; 2 Department of Bioresources, Fraunhofer Institute for Molecular Biology and Applied Ecology, Ohlebergsweg 12, 35392 Giessen, Germany; Tim.Lueddecke@ime.fraunhofer.de (T.L.); michael.marner@ime.fraunhofer.de (M.M.); elena.maiworm@ime.fraunhofer.de (E.M.); johanna.eichberg@ime.fraunhofer.de (J.E.); kornelia.hardes@ime.fraunhofer.de (K.H.); till.schaeberle@ime.fraunhofer.de (T.F.S.); andreas.vilcinskas@ime.fraunhofer.de (A.V.); 3 LOEWE Centre for Translational Biodiversity Genomics (LOEWE-TBG), Senckenberganlage 25, 60325 Frankfurt, Germany; 4 BMBF Junior Research Group in Infection Research “ASCRIBE”, Ohlebergsweg 12, 35392 Giessen, Germany; 5 Institute for Insect Biotechnology, Justus-Liebig-University of Giessen, Heinrich-Buff-Ring 26–32, 35392 Giessen, Germany; 6 German Center for Infection Research (DZIF), Partner Site Giessen-Marburg-Langen, Ohlebergsweg 12, 35392 Giessen, Germany

**Keywords:** pseudoscorpion venom, antimicrobial peptides, antimicrobial activity, cytotoxic activity, insecticidal activity, checacin, megicin

## Abstract

Linear cationic venom peptides are antimicrobial peptides (AMPs) that exert their effects by damaging cell membranes. These peptides can be highly specific, and for some, a significant therapeutic value was proposed, in particular for treatment of bacterial infections. A prolific source of novel AMPs are arthropod venoms, especially those of hitherto neglected groups such as pseudoscorpions. In this study, we describe for the first time pharmacological effects of AMPs discovered in pseudoscorpion venom. We examined the antimicrobial, cytotoxic, and insecticidal activity of full-length Checacin1, a major component of the *Chelifer cancroides* venom, and three truncated forms of this peptide. The antimicrobial tests revealed a potent inhibitory activity of Checacin1 against several bacteria and fungi, including methicillin resistant *Staphylococcus aureus* (MRSA) and even Gram-negative pathogens. All peptides reduced survival rates of aphids, with Checacin1 and the C-terminally truncated Checacin1^1−21^ exhibiting effects comparable to Spinosad, a commercially used pesticide. Cytotoxic effects on mammalian cells were observed mainly for the full-length Checacin1. All tested peptides might be potential candidates for developing lead structures for aphid pest treatment. However, as these peptides were not yet tested on other insects, aphid specificity has not been proven. The N- and C-terminal fragments of Checacin1 are less potent against aphids but exhibit no cytotoxicity on mammalian cells at the tested concentration of 100 µM.

## 1. Introduction

One of the more prevalent medical challenges of our time is the emerging resistance of pathogenic bacteria to antibiotics. In the EU more than 33,000 deaths per year are attributed to infections with multi-resistant bacteria [1]. A possible strategy to address this issue is the discovery of novel compounds with antimicrobial activity. In this regard, antimicrobial peptides (AMPs) are a promising substance class. In contrast to established antibiotics, which normally act on specific cellular processes, AMPs usually exert their effect by disrupting the cell membrane of their target [2]. The specificity of these peptides depends, inter alia, on net charge and hydrophobicity [2] and many exhibit not only antimicrobial but also cytotoxic activities, which can hamper successful drug development [3].

A rich source for the discovery of novel AMPs are animal venoms. This has already been demonstrated by the discovery of many AMPs in the venom of, e.g., spiders, scorpions, snakes, insects, and cone snails [4,5,6,7,8,9,10]. Many of the AMPs discovered in venoms were found to be effective against a wide range of bacteria, including Methicillin-resistant Staphylococci e.g. [11,12]. For some AMPs, an apparent potential for the application against multi-resistant bacteria was already described. These AMPs include marcin and its orthologs from scorpion venom, which were effectively used to treat mice infected with multi-resistant bacteria [13]. Interestingly, the main reasons for rejecting commercial development of scorpion AMP-based antibiotics were rather cost-related or the lack of improvement in efficiency compared to conventional antibiotics, but not the frequent problem of cytotoxicity against human cells [7]. Another application of these compounds is their use as specific pesticides. For instance, oral administration of AMPs from scorpion venom was shown to effectively reduce the number of pea aphids within three days by killing the aphid’s symbiotic bacteria [14]. Hence, AMPs might be promising candidates for alternative pesticides against aphids, which belong to the most important agricultural pests [15]. To increase the chances of identifying novel and highly efficient AMPs, sampling across a wider taxonomic range is advisable.

One group of venomous animals that has been poorly studied regarding its venom composition but has recently been proposed as a potential priority group for bioprospection is the order of pseudoscorpions. Pseudoscorpions, for which more than 3600 species [16] were described, belong to the arachnids and thus share a common ancestor with other venomous lineages, such as spiders and scorpions [17]. Although the more prominent venomous arachnids usually overshadow the pseudoscorpions in the general perception, they nevertheless represent an important group for bioprospection [18]. Within these tiny arthropods, which mostly do not exceed a body length of a few millimeters, a unique venom delivery system has evolved: members of the suborder Iocheirata possess pedipalps equipped with a venom delivery system for subduing their prey. Knowledge about the venom composition of these animals is still limited to a very few species. In the past, the small size and associated handling problems, as well as the miniscule venom yield, have made a proteomic analysis of the venom particularly difficult. Initial studies comprised solely transcriptomic analyses, which provided first insights on the potential venom composition of two pseudoscorpion species [19,20]. However, the possibility of false positives cannot be ruled out when de novo transcriptomic approaches are solely used for the inference of venom compositions [21]. Very recently, a method for extracting pseudoscorpion venom has been developed and thus the implementation of proteomic approaches for studies on pseudoscorpion venom became possible. This enabled for the first time a comprehensive proteo-transcriptomic analysis of the venom composition of the house pseudoscorpion *Chelifer cancroides* [22,23]. These works also led to the discovery of checacins, the first potential AMPs identified from the venom of pseudoscorpions [22]. Checacins were classified as potential AMPs based on their similarity to megicin, an antimicrobial peptide found in the venom of the scorpion *Mesobuthus gibbosus* [24]. As high therapeutic potential has been described for megicin and orthologous sequences [13], checacins might also have beneficial properties in this regard. In our study, we examined the activity of Checacin1 and some Checacin1 fragments in different assays. First, we tested their antimicrobial activity on different strains of Gram-negative and Gram-positive bacteria as well as on different fungi. We also determined the cytotoxicity of the tested peptides to mammalian cells. Finally, their potential for use as pesticides was investigated using aphid feeding assays.

## 2. Results

**Figure 1:**
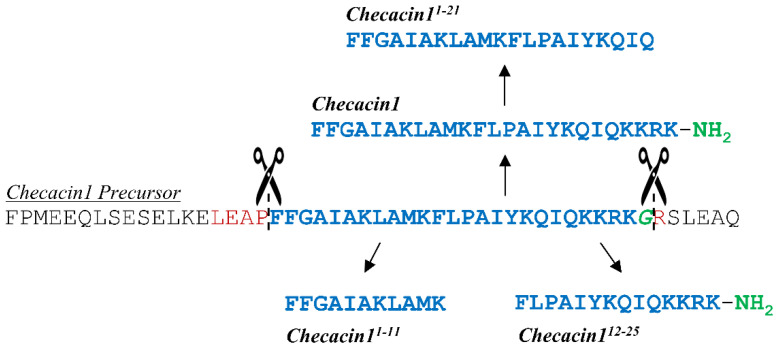
Precursor sequence of Checacin1 (without signal peptide) and the sequences used for the bioassays. Native Checacin1 is N-terminally cleaved at a highly effective ‘LEAP’ motif [22], which is typical of most checacin precursors [23]. The C-terminal cleavage of the Checacin1 progenitor is upstream of a monobasic (Arg) cleavage site and has a C-terminal amidation site (Gly). The preceding KKRK motif does not function as a cleavage signal [22]. Checacin1^1−11^ and Checacin1^12−25^ are naturally occurring fragments of Checacin1, while Checacin1^1−21^ has not been detected in the venom of *C*. *cancroides* [22]. However, for the orthologous Megicin 18 from the scorpion *M*. *gibbosus*, the C-terminal cleavage was postulated to be upstream of such a tetrabasic motif [24].

An overview of the peptides tested in the bioassays is given in Figure 1. In the venom of *C. cancroides*, Checacin1 is highly abundant, whereas the N- and C-terminal fragments Checacin1^1−11^ and Checacin1^12−25^ occur naturally, but with lower abundance [18].

### 2.1. Checacin1 Is Highly Active against Bacteria and Fungi

The minimum inhibitory concentration (MIC) of Checacin1 and the truncated Checacin1 fragments was determined for a diverse panel of Gram-negative and -positive bacteria as well as two fungal indicator strains (Table 1). In order to explore the translational potential of checacins regarding the development of antibiotics, clinically relevant microorganisms were included in the screening. Among these are methicillin-resistant *Staphylococcus aureus* (MRSA) and most importantly *Pseudomonas aeruginosa* as well as *Escherichia coli*. *Mycobacterium smegmatis* was screened as surrogate test organism for *Mycobacterium tuberculosis*. Yeasticidal efficacy was determined using *Candida albicans* as a surrogate strain for *Candida auris*. Only full-length Checacin1 and the C-terminally truncated Checacin1^1−21^ (only on *S. aureus*) exhibited an inhibitory effect on the tested organisms. Checacin1 inhibited the growth of all tested microbes, with the lowest MIC values found for *E*. *coli* and MRSA. No difference of Checacin1 potency towards *E. coli* could be observed when tested in bicarbonate supplemented medium (CAMH-C). With a MIC of 6.25 µM, *C*. *albicans* is notably affected by application of Checacin1, while the growth of the filamentous ascomycete *Aspergillus flavus* was not inhibited.

### 2.2. Orally Administered Checacin1 Is Active against Pea Aphids (Acyrthosiphon pisum)

**Figure 2:**
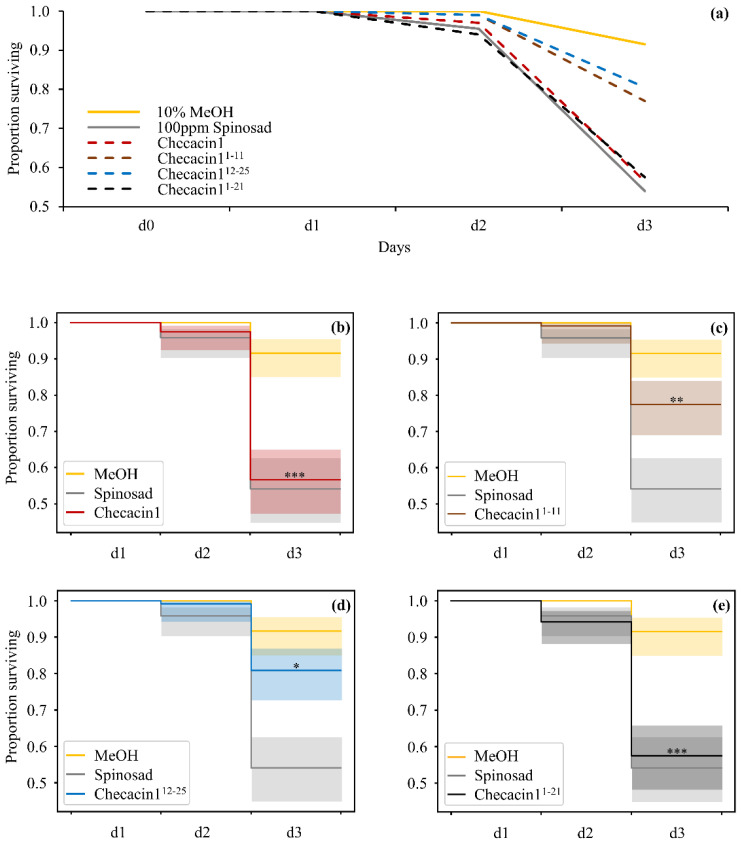
Survival curves of pea aphids (*A. pisum*) after oral administration of Checacin1 and its fragments. Aphids were monitored for three days. As negative control, aphids were fed with 10% methanol (MeOH) and a commercial pesticide was used as positive control (Spinosad). (**a**) Line chart comparing mean survival rates of all tested components with negative and positive control. Checacin1 and Checacin1^1−21^, but not the shorter Checacin1 fragments, were recovered as insecticidal (**b**–**e**) Survival rates of individual components with included 95% confidence interval. Significant differences to the negative controls were assessed by a log-rank test and are indicated by ‘*’ for *p* < 0.1, ‘**’ for *p* < 0.05 and ‘***’ for *p* < 0.01 (Table S1).

To evaluate aphid survival after oral application of Checacin1, aphids were monitored for three days. The aphid survival is compared to application of MeOH (negative control) and the pesticide Spinosad (positive control) in a survival curve (Figure 2, Table S1). Checacin1 and, to lesser degree also Checacin1^1−21^, caused a rapid decrease of the aphid survival rate similar to that of Spinosad (Figure 2a,b). Both killed about half of the aphids (54% and 58% respectively). For the Checacin1 fragments (Checacin1^1−11^, Checacin1^12−25^; Figure 2d,e), the effect on aphid survival is much less distinct compared to the negative control, but still significant.

### 2.3. Cytotoxic Activity of Checacin1

**Figure 3:**
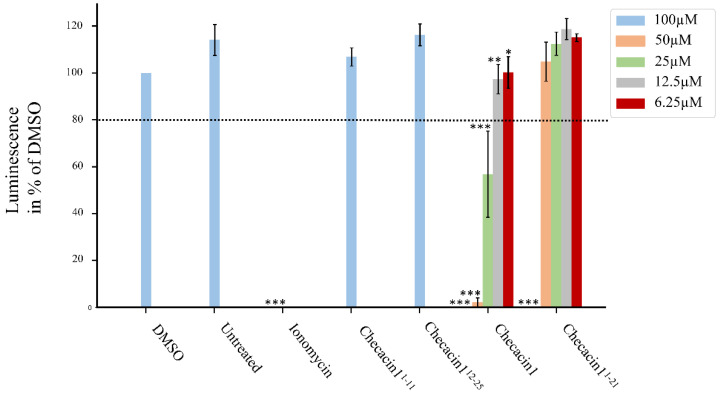
Cell viability assay of MDCK II cells treated with varying concentrations of Checacin1 and Checacin1 fragments. Cell viability was assessed based on CellTiter–Glo^®^ (Promega GmbH, Walldorf, Germany) which measures luminescence as an indicator for ATP amount. The luminescence signal of the 100 µM treatment of Checacine1 and Checacin1^1−21^ was at 0% and therefore not detectable. Luminescence was normalized to the DMSO control; data are presented as mean ± SD (*n* = 4–6). DMSO: dimethyl sulfoxide. Significant differences to the negative control (Untreated) were assessed by t-statistics and are indicated by ‘*’ for *p* < 0.1, ‘**’ for *p* < 0.05 and ‘***’ for *p* < 0.01.

**Figure 4:**
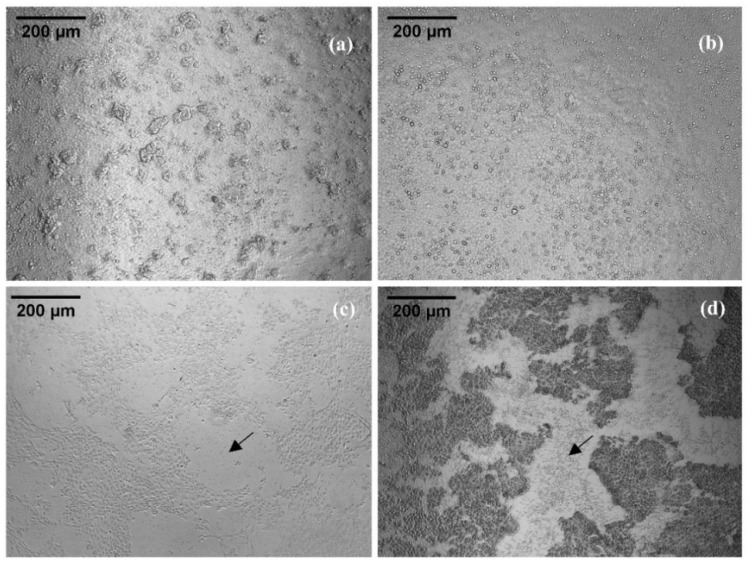
Madin–Darby canine kidney II (MDCK II) cell culture after application of 100 µM Checacin1 and its truncated forms. (**a**,**b**): intact cell layer after application of Checacin1^1−11^ and Checacin1^12−25^ (**c**,**d**): disintegrated cell layer after application of Checacin1^1−21^ and Checacin1. Arrows indicate areas of disintegrated cell layers.

For assessing the cytotoxicity of Checacin1, a CellTiter-Glo^®^ cell viability assay was conducted (Figure 3, Table S2). Cytotoxic effects on the MDCK II cells were mainly observed for Checacin1. Whereas Checacin1^1−11^ and Checacin1^12−25^ caused no visible effects to the cell culture (Figure 4a,b) at the highest applied concentration (100 µM), Checacin1 and C-terminally truncated Checacin1^1−21^ caused a nearly complete disintegration of the cell layer (Figure 4c,d). This was confirmed for both peptides by the significantly lower luminescence compared to the negative control. This is indicative of a strong decline of the cell’s ATP content (Figure 3). For Checacin1, significant cytotoxic effects could also be demonstrated at the lower concentrations 50 µM and 25 µM, however with a relatively high standard deviation in case of the measured luminescence for the 25 µM concentration. For C-terminally truncated Checacin1^1−21^, a concentration of 50 µM does not cause significant cytotoxic effects anymore.

## 3. Discussion

**Figure 5:**
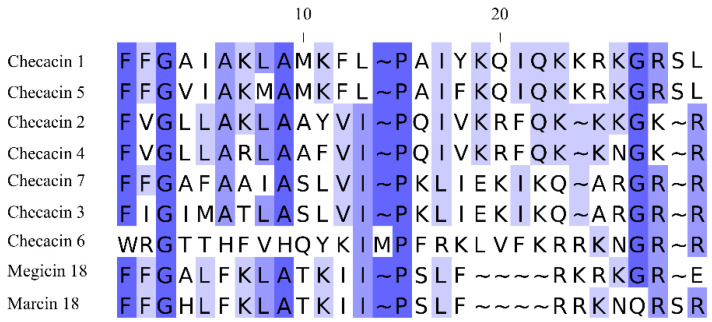
Alignment of the checacin motif, identified in pseudoscorpion venom with orthologs discovered in scorpion venom. Sequences include glycin as amidation signal (if present) and the C-terminal cleavage site. The coloration indicates percentage of identity.

This study provides first insights into the bioactivity of checacins, AMPs identified in the venom of the pseudoscorpion *C*. *cancroides*. By now, precursors of seven checacin genes were identified from this species [22,23]. These checacin genes show highly different expression levels which is also reflected by the relative intensities of the ion signals of the mature peptides in MALDI-TOF mass fingerprints. For our activity tests, we selected Checacin1, which is the most prominent checacin in terms of expression level as well as signal intensity in MALDI-TOF mass spectra of venom samples [22]. Additional peptides used in this study are truncated forms of Checacin1. Two of these peptides represent naturally occurring fragments of Checacin1, whereas synthetic Checacin1^1−21^ is a peptide that was not yet found in the venom of *C. cancroides*. This peptide was tested because AMPs from orthologous scorpion genes (megicin, marcin etc.) were described without the existing basic C-terminus [20]. These peptides have already been synthesized and activity tests resulted in promising antimicrobial activities, including a successful therapy of mice infected with multi-drug-resistant Gram-positive bacteria [13]. Figure 5 shows an alignment of partial checacin precursors from *C. cancroides* with those of partial precursors of the scorpion AMPs megicin and marcin.

As many AMPs found in arachnid venoms, checacins are linear cationic peptides. Such peptides mostly exert their toxic effects by binding to cell membranes and causing the formation of pores, leading to cell death [25]. A crucial factor for the pore forming ability is the hydrophobic N-terminus, whereas the specificity for different membranes is mainly determined by the net charge of the peptide [25]. As bacterial cell membranes exhibit a more negative net charge than, e.g., animal cell membranes, cationic peptides bind to these with higher affinity [26]. In the venom, such peptides were first believed to function solely as conserving agents which protect the venom peptides against a wide range of microorganisms [27]. As linear cationic peptides often also exhibit cytotoxic activities on the potential prey, an active participation as, e.g., spreading factors [28] in envenomation cannot be excluded and the already postulated ‘dual-use’-concept [29] might be the best approximation to explain the mode of action of the majority of these peptides.

In our tests, antimicrobial activities were only observed for full length Checacin1 and in part for Checacin1^1−21^. The highest applied concentrations of the shorter checacin fragments did not cause any antimicrobial effects on the tested strains. This can be explained by the following assumptions: in case of Checacin^1−11^, the lower net charge reduces antimicrobial activity, whereas Checacin1^12−25^ is lacking the hydrophobic N-terminus. Checacin1^1−21^ is also much less active against the tested strains and only efficient on *S*. *aureus*. As this peptide lacks the C-terminus, the reduced antimicrobial activity might also be explained by the reduced positive net charge. The C-terminal amidation improves peptide stability by protecting the peptide from proteolytic cleavage. In the case of the checacin precursors, the use of the monobasic Arg cleavage signal following Gly may have been subject to positive selection during the evolution of the checacins. It was shown in a previous study, that synthetic scorpion AMPs are effective against aphids by acting on their symbiotic bacteria [14]. Interestingly, both Checacin1 and Checacin1^1−21^ killed aphids with similar efficiency, though the antimicrobial efficacy of Checacin1^1−21^ is much weaker. The N- and C-terminal fragments of Checacin1 also had a weak but significant effect on aphid survival without noticeably affecting any of the microbes tested. This indicates that at least some of the tested Checacin1 fragments exert their insecticidal activity on aphids by directly affecting the aphid cells and not the symbiotic bacteria. Nevertheless, the aphid’s symbionts were not yet among the tested strains, and the peptide concentrations used in the aphid assay were mostly higher than the concentrations applied in the antimicrobial tests. Regarding the cytotoxicity for mammalian cells, mainly Checacin1 showed cytotoxic effects in the tested concentration range. Cytotoxic effects caused by AMPs were observed frequently in the past, e.g., [21]. Interestingly, the mechanism by which AMPs act on most mammalian cells is usually apoptosis and not cell lysis as observed for bacterial cells and erythrocytes [30]. A requirement for pharmaceutic application of AMPs is a MIC lower than the minimal concentration causing cytotoxic effects in mammals [31]. In case of Checacin1, the minimal cytotoxic concentration is between 25 µM and 12.5 µM, which is one order of magnitude above the observed MICs of *E*. *coli* and *S*. *aureus*. Due to this minimal therapeutic window, pharmaceutical application would require further optimization. However, in the current study cytotoxicity was tested with MDCK II cells from dogs and the effect on human cells has still to be examined. Bacalum and Radu (2015) recommended to test cytotoxicity on erythrocytes and lymphocytes to consider apoptotic effects caused by AMPs [30]. A yet unresolved question regarding differential expression of the checacins is to what extent the relative abundance of the checacins correlates with specificity or efficiency toward their target cells. In this regard, three hypotheses seem plausible: (1) The highly abundant checacins are the most efficient, which would explain the higher energy investment of increased production. (2) Less abundant checacins are more efficient and due to their higher efficacy, higher concentrations are not necessary to fulfil their functions. It has been postulated previously, that venoms contain toxins with low abundance, that are equally important as the products of highly expressed venom compounds e.g., [32]. (3) The ratio of checacins in the venom might lead to an optimal efficiency. This idea might be supported by findings that demonstrated synergistic effects of venom compounds, e.g., [33].

## 4. Conclusions

The venom of neglected arthropods provides an additional source of novel AMPs, which are promising candidates for developing alternative pesticides or antibiotics. For full-length Checacin1, the most prominent AMP present in the venom of the pseudoscorpion *C. cancroides*, we observed promising antimicrobial activities against several clinically relevant strains of bacteria and fungi. In feeding assays, Checacin1 and C-terminal truncated Checacin1^1−21^ efficiently reduced aphid survival, suggesting that these peptides have potential as novel pesticides, although their selectivity for pest insects needs to be explored further. As known for linear cationic peptides, the cell membrane disrupting capability of Checacin1 relies on its hydrophobic N-terminus as well as on the high net charge, which is demonstrated by the much lower efficacy observed for Checacin1 fragments. So far, we have only tested the activity of one out of seven known *C. cancroides* checacins. As these peptides differ substantially in terms of gene expression level, it would be interesting to test, how this is correlated with the efficiency and specificity of the different checacins.

## 5. Material and Methods

### 5.1. Peptide Synthesis

Synthetic peptides were obtained from DGpeptides Co., Ltd. (Hangzhou, China). Purities and identifiers used by the company are shown in Table 2.

### 5.2. Feeding Assay on Pea Aphids (A. pisum)

Screenings for potential insecticidal activities were performed as feeding assays on age synchronized pea aphids (*A. pisum,* clone LL01, 5 days old) as described by Heep and colleagues [34,35]. Pea aphid nymphs were fed on an artificial diet [36], containing the tested checacins (100 ppm), in specialized feeding chambers [37] for three days. We used diet mixtures containing 10% methanol as negative control and Spinosad (100 ppm) as positive control. Pea aphid survival was scored daily. The feeding assay was performed in three biological replicates per substance and control, each containing a total of 60 *A. pisum* specimen.

### 5.3. Antimicrobial Activity Assays

The antimicrobial properties of the synthesized checacins were evaluated against a diverse set of indicator strains. The used method for minimum inhibitory concentrations (MIC) determination is derived from the methodology proposed by the EUCAST committee. The antibacterial/antifungal MIC value is defined as the lowest concentration of an agent that inhibits the growth of a microorganism by 85% relative to the growth controls (High value). The medium background (low value) is subtracted from all measurements (*AU* = absorption units; luminescence for cell viability assays). Relative growth inhibition is calculated according to:

$$rel.growth\;inhibtion\left[\%\right]=100\;\times \;\left(1-\frac{AU_{sample}-\;AU_{Low}\;}{AU_{High}-\;AU_{Low}}\right)$$

*AU = absorption units, low = medium blank, high = growth control*.

The peptides were dissolved in sterile ultra-pure water (0.055 μS/cm) and tested in a 12 step dilution series ranging from 50 to 0.02 μM. All concentrations were tested in triplicate.

*Escherichia coli* ATCC35218, *Staphylococcus aureus* ATCC33592 MRSA and *Pseudomonas aeruginosa* ATCC27853 were incubated overnight (37 °C, 180 rotations per minute (rpm)) and subsequently diluted to 5 × 10^5^ cells/mL in cation adjusted Mueller Hinton II medium (Becton Dickinson, Sparks, NV, USA). For *E. coli* ATCC35218 an additional cell suspension was prepared in cation adjusted Mueller Hinton II medium supplemented with 44 mM sodium bicarbonate. As positive controls dilution series of rifampicin, tetracycline and gentamycin (all Sigma Aldrich, St. Louis, MS, USA), ranging from 64–0.03 μg/mL, were used. Bacterial suspensions without peptide or antibiotic control were used as negative controls. After assay incubation (37 °C, 180 rpm, 85% relative humidity (r.H.)), cell growth was assessed by measuring the turbidity with a microplate spectrophotometer at 600 nm (LUMIstar^®^ Omega BMG Labtech, Ortenberg, Germany). Growth inhibition was calculated relative to the absorption of the controls. The pre culture of *Mycobacterium smegmatis* ATCC607 was incubated in brain–heart infusion broth (Becton Dickinson, Sparks, NV, USA) for 48 h, at 37 °C and 180 rpm before the cell concentration was adjusted in Mueller Hinton II medium. Isoniazid (Sigma Aldrich, St. Louis, MS, USA) was used instead of gentamycin as third positive control. Cell viability was evaluated after 48 h (37 °C, 180 rpm, 85% r.H.) via ATP quantification (BacTiter-Glo™, Promega, Madison, WI, USA) according to the manufacturer’s instructions. *Candida albicans* FH2173 was incubated for 48 h at 28 °C and 180 rpm before the pre culture was diluted to 1 × 10^6^ cells/mL in Mueller Hinton II medium. For *Aspergillus flavus* ATCC9170, a previously prepared spore solution was used to adjust the assay inoculum to 1 × 10^5^ spores/mL. Assays were incubated for 48 h at 37 °C and 180 rpm. For both, tebuconazole (Cayman Chemical Company, Ann Arbor, MI, USA), amphotericin B and nystatin (both Sigma Aldrich, St. Louis, MS, USA) were used as a positive control (64–0.03 μg/mL). Readout was performed by way of ATP quantification.

### 5.4. Cytotoxicity Assays

#### 5.4.1. Cell Culture

Madin–Darby canine kidney II (MDCK II) cells [38] were maintained in Dulbecco’s modified Eagle’s medium (DMEM GlutaMAX, ThermoFisher, Waltham, MA, USA) supplemented with 1 % penicillin/streptomycin (ThermoFisher) and 10 % fetal calf serum (ThermoFisher) and grown in an incubator at 37 °C with 5 % CO_2_. The MDCK II cell line was kindly provided by Prof. Dr. Friebertshäuser (Philipps University Marburg, Institute of Viorology, Marburg, Germany).

#### 5.4.2. Cell Viability Assays

Checacins and Ionomycin (Cayman Chemicals, Ann Arbor, MI, USA) were dissolved in DMSO and Aprotinin (Carl Roth, GmbH & co KG, Karlsruhe, Germany) was dissolved in water (stock solutions: 10 mM).

For the cell viability assay MDCK II cells were seeded in a 96-well plate and treated at a confluence of 90% with the indicated inhibitors (100 μM per well) or DMSO control. The plate was incubated at 37 °C and 5% CO_2_. The cell viability was assessed by measuring the ATP content using the CellTiter-Glo Luminescent Cell Viability assay (Promega GmbH, Walldorf, Germany) according to the manufacturer’s instructions. Luminescence was measured using a black 96-well plate in a Synergy H4 microplate reader (Biotek, Waldbronn, Germany, now part of Agilent Technologies). Relative light units (RLU) were normalized to DMSO control set to 100%. Measurements were conducted with four to six replicates and standard deviations were calculated.

## Figures and Tables

**Table 1:** Minimum inhibitory concentrations (MIC) of Checacin1 and Checacin1 fragments determined for different microbes (*Ec*: *Escherichia coli*, *Pa*: *Pseudomonas aeruginosa*, *Ms*: *Mycobacterium smegmatis*, *Sa*: *Staphylococcus aureus*, *Af*: *Aspergillus flavus*, *Ca*: *Candida albicans*). CAMH-II: cation-adjusted Mueller Hinton II medium; CAMH-C: CAMH-II with 44 mM sodium bicarbonate; TEM-1: TEM-1 beta-lactamase expressing strain; MRSA: methicillin-resistant *Staphylococcus aureus*; BTG: BacTiter-Glo™-assay; MTT: microtiter turbidity assay.

Compounds	MIC (µM)						
	***Ec***		***Pa***	***Ms***	***Sa***	***Af***	***Ca***
	ATCC 35218		ATCC27853	ATCC607	ATCC33592	ATCC9170	FH2173
	TEM-1	TEM-1			MRSA		
	CAMHII	CAMH-C	CAMHII	CAMHII	CAMHII	CAMHII	CAMHII
Checacin1	1.6–0.8	1.6	12.5	25	1.6	50	6.25
Checacin1^1−11^	>50	>50	>50	>50	>50	>50	>50
Checacin1^12−25^	>50	>50	>50	>50	>50	>50	>50
Checacin1^1−21^	>50	>50	>50	>50	12.5	>50	>50
Rifampicin	4.9	19.4	38.9	38.9–19.4	>77.8	NA	NA
Tetracycline	4.5	36–18	>144.1	0.14	>72	NA	NA
Gentamycin */Isoniazid ’’	2.1–1	1–0.5	1	29.2–14.6 ’’	0.5–0.26	NA	NA
Tebuconazole	NA	NA	NA	NA	NA	0.19	0.19–0.09
Amphotericin B	NA	NA	NA	NA	NA	1.1	8.6–4.3
Nystatin	NA	NA	NA	NA	NA	1.1	8.6
readout	MTT	MTT	MTT	BTG	MTT	BTG	BTG

* Calculated for gentamycin C1 (25876-10-2) as gentamycin (1405-41-0) is a substance mixture. ’’ *Mycobacterium smegmatis* was tested against isoniazid instead of gentamycin.

**Table 2:** List of antimicrobial peptides synthesized based on peptides identified in the venom of the pseudoscorpion *Chelifer cancroides*.

Component/Company ID	Sequence	Purity
Checacin1/D-4040	FFGAIAKLAMKFLPAIYKQIQKKRK *	96.75%
Checacin1^1−11^/D-4041	FFGAIAKLAMK	97.84%
Checacin1^12−25^/D-4042	FLPAIYKQIQKKRK *	98.07%
Checacin1^1−21^/D-4043	FFGAIAKLAMKFLPAIYKQIQ	96.87%

* Sequence with C-terminal amidation.

## Data Availability

Data is contained within the article and the Supplementary Materials.

## Supplementary Materials

The following supporting information can be downloaded at: https://www.mdpi.com/article/10.3390/toxins14010058/s1, Table S1: ‘Aphid Raw Data’, Table S2: ‘Cytotox Raw Data’.
